# Emodin inhibits colon cancer by altering BCL-2 family proteins and cell survival pathways

**DOI:** 10.1186/s12935-019-0820-3

**Published:** 2019-04-15

**Authors:** Ian T. Saunders, Hina Mir, Neeraj Kapur, Shailesh Singh

**Affiliations:** 1 Department of Microbiology, Biochemistry and Immunology and Cancer Health Equity Institute, Morehouse School of Medicine, Atlanta, GA 30310 USA; 2 Cancer Health Equity Institute, Morehouse School of Medicine, Atlanta, GA 30310 USA

**Keywords:** Colon cancer, Emodin, Anthraquinone, Apoptosis, Mitochondria, Bcl-2

## Abstract

**Background:**

Currently offered therapeutics to treat colon cancer (CoCa) are toxic when given at maximum tolerated dose to achieve optimal clinical response. Hence, less toxic therapeutic intervention is needed to treat CoCa. In this study, we investigated the effect of a natural agent, Emodin, on CoCa.

**Methods:**

Cell viability (MTT) assay was used to determine the effect of Emodin on human CoCa and colon epithelial cells. Flow cytometric analysis was used to determine Emodin induced cell death. Antibody microarray and western blot analyses were used to determine Emodin induced molecular changes involved in cell death. Change in mitochondrial membrane potential in response to Emodin was determined by flow cytometric analysis. Expression and localization of Bcl-2 family proteins were assessed by western blot analysis.

**Results:**

Emodin decreased viability of CoCa cells and induced apoptosis in a time and dose-dependent manner compared to vehicle-treated control without significantly impacting normal colon epithelial cells. Emodin activated caspases, modulated Bcl-2 family of proteins and reduced mitochondrial membrane potential to induce CoCa cell death. Further, changes in Bcl-2 family protein expression and localization correlated with loss in mitochondrial membrane potential. Signaling (MAPK/JNK, PI3K/AKT, NF-κβ and STAT) pathways associated with cell growth, differentiation, and Bcl-2 family expression or function were negatively regulated by Emodin.

**Conclusions:**

Ability of Emodin to impact molecular pathways involved in cell survival and apoptosis highlight the potential of this agent as a new and less toxic alternative for CoCa treatment.

## Background

Colorectal cancer is the third most diagnosed and second leading cause of cancer-related deaths in both men and women in the United States [1, 2]. Most cancers, including colon cancer (CoCa), progress by up-regulating cell growth and survival signaling while evading cell death [3–8]. As a result, treatment modalities that not only reduce CoCa cell growth but also more importantly re-establish a proper balance in cell death and survival could be better treatment options. Current treatment options [9] fail to achieve optimal clinical response due to development of resistance and toxicity. Hence, less toxic and efficacious therapy is needed to treat CoCa.

Extensive research to assess the potential of nutrients and herbal extracts as a single agent or as an additive to conventional therapeutics has been conducted in cancer prevention and treatment [10–13]. Biochemical and molecular investigations have demonstrated that several classes of compounds present in vegetables and fruits possess beneficial properties to fight against infection, inflammation, and oxidation [14]. Anthraquinones are a class of natural compounds that exhibit many of these beneficial properties and induce cancer cell death [14–17]. Emodin, an anthraquinone derived from more than 17 plant families worldwide, has been used for centuries as herbal laxative, anti-inflammatory or anti-microbial agent [17–20]. Studies have shown that Emodin promotes apoptosis, inhibits DNA synthesis, and promotes free radical generation in breast, lung, and prostate cancers. Further, this agent has the ability to inhibit metastasis and increase the efficacy of other anti-cancer agents with minimal toxicity to normal cells [20–28]. Many of these anti-cancerous properties have been explored in CoCa, but the mechanistic studies defining the molecular pathways impacted by Emodin have not been fully defined. In this study, the molecular changes associated with Emodin-induced apoptosis, Bcl-2 family gene expression, and survival signaling in CoCa cells were evaluated. Our findings highlight the potential of Emodin as a potent anti-cancer agent in the treatment of advanced CoCa.

## Methods

### Materials

Emodin [1,3,8-trihydroxy-6-methylanthracene-9,10-dione; purity ≥ 95] was procured from LKT Laboratories (Cat#:518-82-1) (St. Paul, MN, USA) and DMSO was purchased from Corning Cellgro (Cat#:MT-25950CQC) (Manassas, VA, USA). For western blot analyses, rabbit derived antibodies specific for Bad (Cat#9239T), Bak (Cat#12105), Bax (Cat#5023), Bim (Cat#2933), total (Cat#9662) and cleaved-Caspase-3 (Asp175) (Cat#9664), cleaved-Caspase-9(Asp315) (Cat#20750), Cytochrome C (Cat#11940), phosphorylated (Thr202/Tyr204) (Cat#4370) and total (Cat#9102) p44/p42 MAPK ERK1/2, p53 (Cat#2527), PARP (Cat#9532), PDK1 (Cat#5662), p-PTEN(Ser380) (Cat#9551), PUMA (Cat#12450), Ras (Cat#3339), STAT-1 (Cat#9172), STAT-3 (Cat#30835S), and anti-rabbit IgG HRP-linked secondary antibody was purchased from CST (Denvers, MA, USA). Mouse derived antibodies specific for Bid (Cat#8762), cleaved (Asp387) (Cat#8592) and total (Cat#9746) Caspase-8, total Caspase-9 (Cat#9508), p-NF-κβ (S380) (Cat#6956), and anti-mouse IgG HRP-linked secondary antibody was purchased from CST (Danvers, MA, USA). β-actin mouse (C4) (Cat#sc-47778) mAb was purchased from Santa Cruz Biotechnology (Dallas, TX, USA). Bcl-2 mouse (Bcl-2-100) (Cat#13-8800) mAb was purchased from ThermoFisher Scientific (Waltham, MA, USA).

### Cell lines and culture conditions

Human CoCa cells (DLD-1 and COLO-20) and normal colon epithelial cells (CCD 841 CoN) were purchased from the ATCC (Rockville, MD, USA). Cells were cultured in RPMI-1640 medium (Cat#MT10040CM) (Corning Cellgro, Manassas, VA, USA) supplemented with 10% FBS, amphotericin B (250 μg/mL), HEPES Buffer (238.3 μg/mL), streptomycin (100 μg/mL) and penicillin (100 U/mL) (Corning Cellgro, Manassas, VA, USA). All cells were cultured at 37 °C with 5% CO_2_ atmosphere. Prior to each experiment, cells were cultured in 2% FBS containing media for 24 h.

### Cell viability

Normal colon epithelial and CoCa cells were seeded (1.5 × 10^4^ cells/well) in triplicate in a 96-well plate for 24 h. Cells were treated with increasing concentrations of Emodin (0,10, 20, 40, and 80 μM), dissolved in DMSO (0.01% final concentration), or vehicle control (0.01% DMSO) for 24, 48, and 72 h. At the conclusion of each time point, 20 μL of MTT reagent (5 mg/mL in PBS) was added to each well and incubated for 2 h to allow formazan crystallization. Media was then discarded and formazan crystals were solubilized in DMSO. Absorbance was measured at 570 nm using a SpectraMax M5 spectrophotometer (Molecular Devices, San Jose, CA, USA). The half maximal effective concentration (EC_50_) of each cell line as determined by a linear regression using GraphPad Prism software (GraphPad Software, La Jolla, CA, USA) was used for subsequent experiments.

### Apoptosis assay

Cells were seeded (10^6^ cells/well) in duplicate in 6-well plates for 24 h and treated with EC_50_ of Emodin or vehicle control (0.01% DMSO) for 24, 48, or 72 h. Cells were harvested, washed twice with ice-cold cell staining buffer and equal number of cells were then stained with FITC-Annexin V and 7-AAD using the Apoptosis Detection Kit (Cat#640922) (BioLegend, San Diego, CA, USA), according to manufacturer’s instructions. The fluorescence of the stained cells was acquired (30,000 events/sample) using a Guava flow cytometer (Millipore, Billerica, MA, USA) and analyzed using FlowJo 10.0.06 software (Treestar Inc., Ashland, OR, USA).

### Antibody microarray

An apoptosis associated antibody microarray (Cat#APP069) (Full Moon BioSystems, Sunnyvale, CA, USA) consisting of 73 highly specific and well characterized antibodies against apoptosis and related survival signaling was used to evaluate effect of Emodin treatment on CoCa cells. Cells were treated with EC_50_ of Emodin or DMSO for 48 h. Protein extracts from all samples were collected and biotinylated (75μg protein/reaction) using the Antibody Array Assay Kit (Cat#KAS02) (Full Moon BioSystems). Samples were coupled with array slides and labeled with 0.5 mg/mL Cy3-Streptavidin as directed in the manufacturer’s instructions. The arrays were scanned for signal quantification by Full Moon BioSystems using an Axon GenePix 4000B microarray scanner (Molecular Devices, Sunnyvale, CA, USA). Each antibody was replicated six times and β-actin and GAPDH were used as internal controls. Average signal intensity of replicate spots was normalized first to the median signal of the slide, then to that internal control. Ratio of intensities of Emodin treated versus untreated samples was used as fold changes in protein expression. Heat map was generated using the CIMminer platform (https://discover.nci.nih.gov/cimminer/oneMatrix.do) developed by the Genomics and Bioinformatics Group at the NCI.

### Mitochondrial membrane potential assay

Cells were seeded (10^6^ cells/well) in 6-well plates for 24 h followed by treatment with Emodin, or vehicle (0.01% DMSO) for 24, 48, or 72 h. Cells were harvested, washed twice with ice cold PBS, resuspended in JC-1 containing assay buffer (Cat#30001) (Biotium Inc., Fremont, CA, USA), and stained as per the manufacturer’s instruction. Cells were then incubated for 15 min at 37 °C with 5% CO_2_, washed and resuspended in PBS. Fluorescence was acquired and analyzed as mentioned before.

### Cytosolic and mitochondrial protein isolation

Cytosolic and mitochondrial protein fractions were extracted from vehicle-treated or Emodin treated CoCa cells using Mitochondria/Cytosol Fractionation Kit (Cat#K256-25) (BioVision Inc, Milpitas, CA, USA). Briefly, 4 × 10^6^ cells were harvested, washed and centrifuged at 600*g*/5 min/4 °C. Cells were resuspended in cytosol extraction buffer mix, incubated on ice for 10 min, and homogenized on ice using Omni TH homogenizer (Omni Inc., Kennesaw, GA, USA). The homogenate was centrifuged at 700*g*/10 min/4 °C. Supernatant was collected and centrifuged at 10,000*g*/30 min/4 °C. That supernatant was used as the cytosolic fraction. The pellet was resuspended in mitochondrial extraction buffer mix, vortexed for 10 s, and used as the mitochondrial fraction.

### Western blot analyses

Total protein from Emodin or vehicle-treated cells was isolated using RIPA buffer containing protease (Thermo Fisher Scientific, Waltham, MA, USA) and phosphatase inhibitors (Millipore Sigma, St. Louis, MO, USA). Protein was quantified by BCA method (Thermo Fisher Scientific, Waltham, MA, USA). Proteins (50 µg/lane) were loaded, resolved on 12% SDS-PAGE, and transferred to PVDF membranes. Membranes were incubated in blocking buffer (5% nonfat milk in PBST i.e. PBS/0.1% Tween-20) for 1 h at RT. Membranes were incubated with specific primary antibodies overnight at 4 °C followed by three washes with PBST, then incubated with respective secondary antibody for 2 h. Primary and secondary antibodies were diluted 1:1000 and 1: 2000, respectively in 3% non-fat milk dissolved in PBST. West Pico or Trident Femto Chemiluminescent substrate (GeneTex Inc., Irvine, CA, USA) was used to develop the bands, and Image J software (http://www.rsbweb.nih.gov/ij) was used to quantify the results.

### Statistical analyses

The statistical significance between different groups was determined either by Student’s *t* test or by two-way ANOVA followed by Holm-Sidak’s post hoc test for multiple comparisons using GraphPad Prism version 6.0 (GraphPad Software Inc., La Jolla, CA, USA). The graphs presented are representative of three independent experiments. Statistical significance was established at p < 0.05 and results are shown as mean ± SEM.

## Results

### Emodin reduces the cell viability of human colon cancer cells

**Fig. 1:**
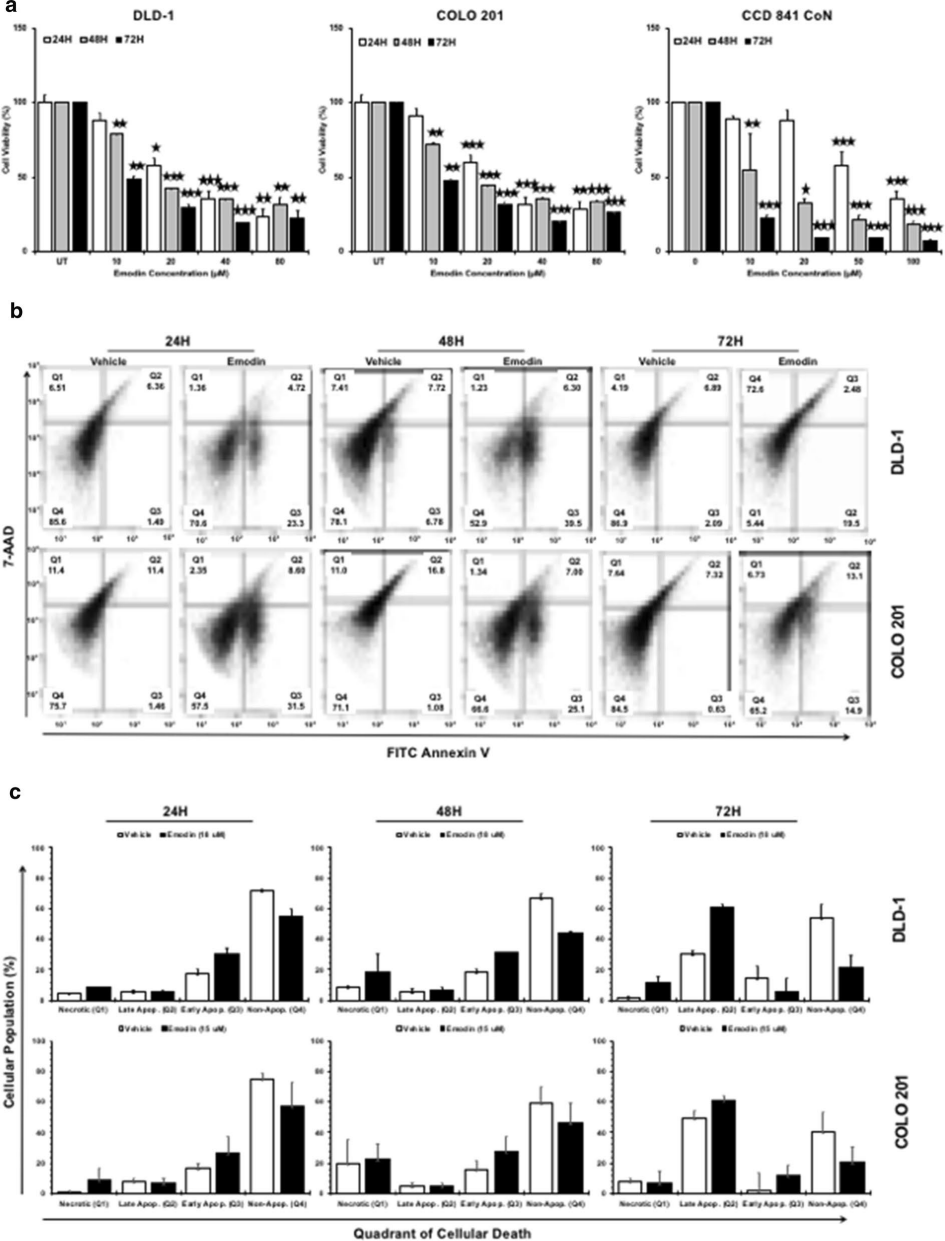
Emodin reduces the cell viability and induces apoptosis in human colon cancer cells. Colon epithelial cells (CCD 841 CoN) and colon cancer (CoCa) cells (DLD-1 and COLO 201) were treated with increasing concentrations of Emodin (0–80 μM) for 24 h (white bar) 48 h (grey bar) and 72 h (black bar). MTT assay was used to measure cell viability and EC_50_ was determined via a linear regression (**a**). DLD-1 and COLO 201 cells were treated with EC_50_ of Emodin 18 μM and 15 μM, respectively and apoptosis was assessed by staining with FITC-conjugated Annexin V and 7-AAD (**b**). The percentage of necrotic, late and early apoptotic and non-apoptotic cells are shown in Q1, Q2, Q3 and Q4. **c** Bar diagrams show percentage changes in necrotic, late and early apoptotic, and non-apoptotic cells as mean ± SEM from three independent experiments. Asterisks (*) p < 0.05, (**) p < 0.01, (***) p < 0.001, show significant change compared with the control

A dose- and time-dependent decrease in the viability of CoCa cells occurred following Emodin treatment (Fig. 1a). Treatment with as low as 10 μM Emodin led to significant decrease in CoCa cell viability at 48 h (DLD-1 21%, p < 0.001; COLO 201 28%, p < 0.01) and 72 h (DLD-1 51%, p < 0.01; COLO 201 52%, p < 0.01). When compared to the control, at 24 h, a ~ 40% reduction in cell viability (DLD-1 cells, 41%, p < 0.01; COLO 201, 40%, p < 0.001) was observed with 20 μM Emodin treatment of CoCa cells. At 48 h, 20 μM Emodin treated CoCa cells showed a greater reduction in the cell viability (DLD-1: 57%, p < 0.001, COLO 201: 55%, p < 0.001). Cell viability of CoCa cells, following 20 μM Emodin treatment, was reduced remarkably at 72 h (DLD-1 70%, p < 0.001; COLO 201 68%, p < 0.001). Such trend of reduction in viability was seen at all higher concentrations of Emodin at all three time points. However, effect of Emodin on normal epithelial cell viability was minimal. It decreased CCD 841 CoN viability at 24 h by 50% at concentrations higher than 50 μM. Emodin treatment at 48 h reduced cell viability to < 50% at concentrations above 10 μM. CCD 841 CoN cell viability was reduced to ~ 20% with 10 μM treatment at 72 h.

All consequent experiments were done using 48 h EC_50_ which was found to be ~ 18 μM for DLD-1 (42% viability) and ~ 15 μM for COLO-201 (44% viability).

### Emodin induces apoptosis in human colon cancer cells

To determine CoCa cell death induced by Emodin, a FITC-Annexin V/7-AAD assay was conducted. Annexin-V binds with high affinity to extracellular phosphatidylserine (PS) during apoptosis and 7-AAD exclusively binds DNA of apoptotic cells identifying stages of cell death following treatment (Fig. 1b, c). The percentages of cells in treated minus percentages of cells in untreated groups, as shown in parentheses, were used to infer the data. At 24 h, number of CoCa cells in early apoptotic (Annexin V-positive and 7-AAD-negative) phase (22% DLD-1; 30% COLO 201) increased after Emodin treatment compared to control. This trend continued after 48 h with higher number of early apoptotic cells (33% DLD-1; 24% COLO 201) in Emodin treated group compared to control. Interestingly, we did not see a significant shift in cells that were positive for both Annexin V and 7-AAD (late apoptotic population) when compared to control. The most significant increase (11% DLD-1; 7% COLO 201) in late apoptotic cells was seen after 72 h Emodin treatment. COLO 201 cells had a 15% increase in early apoptotic cells which could account for the small increase in late apoptotic population. These findings show that Emodin affects CoCa cell growth mainly by inducing apoptosis.

### Emodin modulates the expression of anti- and pro-apoptotic molecules to induce cell death in colon cancer

**Fig. 2:**
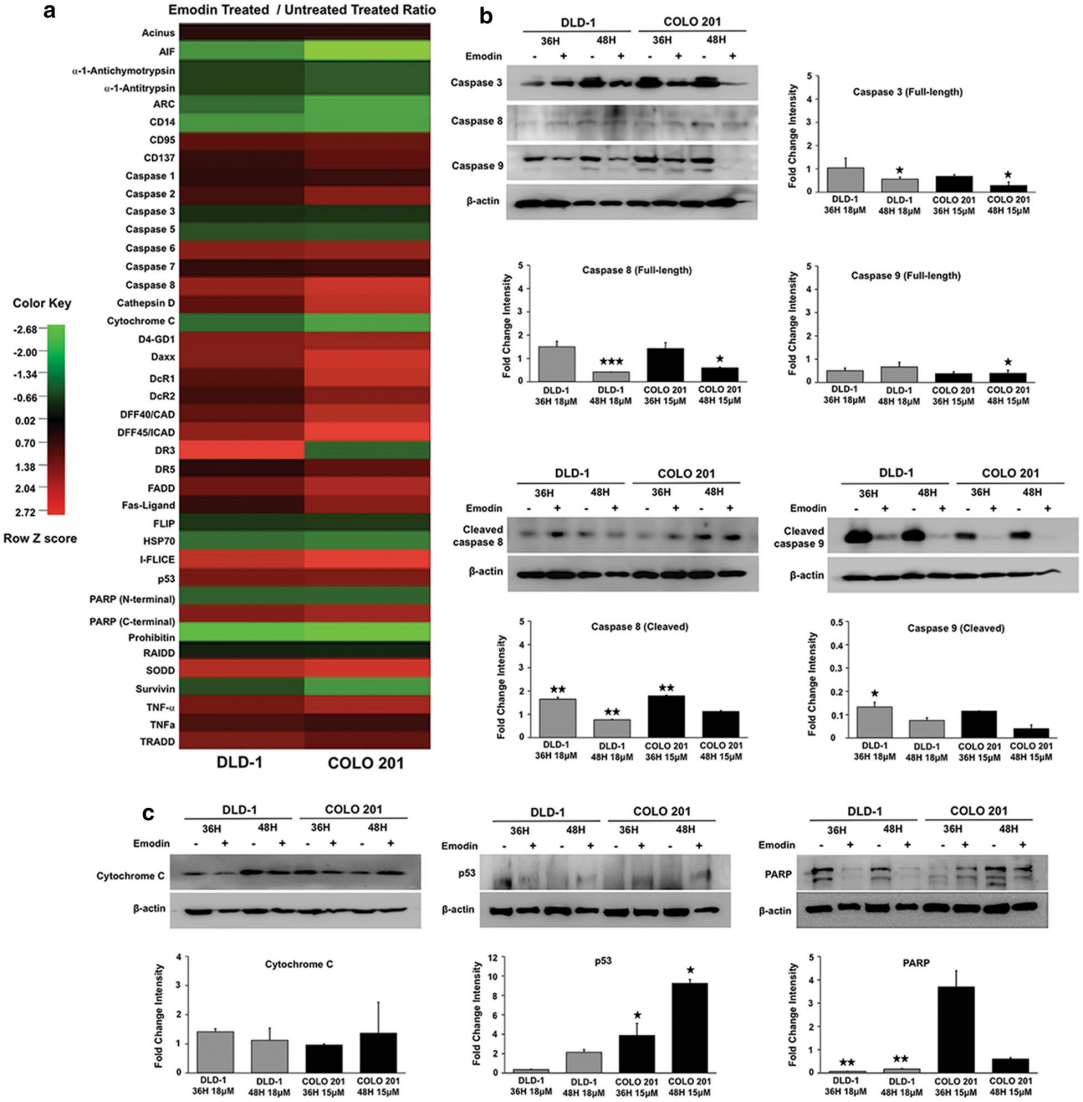
Emodin induces colon cancer cell death by modulating apoptotic proteins. Apoptosis antibody microarray was probed with protein from Emodin-treated (DLD-1 ~ 18 μM; COLO 201 ~ 15 μM; 48 h) and vehicle-treated CoCa cells and heatmap was generated from normalized intensity data using CIMminer. Each cell in the heatmap represents the fold change in apoptosis-related protein in Emodin-treated CoCa cells compared to vehicle-treated control. Red represents increase while green represents decrease in protein expression, and intensity of color indicates expression levels (**a**). Changes in Caspase (**b**),  Cytochrome C, p53 and PARP expression (**c**) after Emodin treatment are shown in representative western images. β-actin was used as the loading control. The quantitative bar graphs in panel (**b**) and (**c**) represent ratio of protein in Emodin treated versus vehicle-treated after normalization with house keeping protein intensity. All graphs represent the mean fold change intensity value ± SEM from three independent experiments; Asterisks (*) p < 0.05, (**) p < 0.01, (***) p < 0.001 show significant change in response to Emodin treatment compared with the control

Antibody microarray analysis showed significant changes in caspase activity in response to Emodin treatment. Initiator caspase-8 expression increased (DLD-1: ~ 1.5-fold; COLO 201: ~ 2.3-fold) in both CoCa cells (Fig. 2a) whereas caspases-3 and -5 expression decreased (caspase-3 ~ 0.6 fold; caspase-5 ~ 0.8 fold) and caspase-1, -2, -6, and -7 increased in both cell lines after Emodin treatment (Fig. 2a). Antibody microarray results were further confirmed by western blots (Fig. 2b). The quantitative graphs represent ratio of protein in Emodin treated versus vehicle-treated after normalization with house keeping protein intensity. Cleaved caspase 8 levels increased at 36 h in both cell lines following Emodin treatment, but decreased at 48 h (DLD-1: p < 0.01) in both cell lines as compared to vehicle control. Significant decrease in full-length caspase-8 at 48 h (DLD-1: p < 0.0001; COLO 201: p < 0.05) in both cell lines as compared to vehicle-treated control further support the observed caspase 8 activation. Full-length caspase-9 significantly decreased at 48 h in COLO 201 cells (p < 0.05) after treatment compared to control. Cleaved caspase-9 levels significantly decreased at 36 h in DLD-1 cells (p < 0.05) compared to control. Full-length caspase-3 levels significantly decreased (p < 0.05) in both cells at 48 h, confirming the antibody array data (Fig. 2a).

In addition to caspases, antibody microarray (Fig. 2a) and western blot analysis (Fig. 2c) show a significant increase in p53 and decrease in PARP protein levels in response to Emodin treatment. There was ~ 1.6 fold increase in p53 protein in both cell lines in the array (Fig. 2a). Western blot showed significant (p < 0.05) increase in p53 in COLO 201 cells at 36 and 48 h compared to control. However, in DLD-1 an increase in p53 expression was only observed at 48 h, which was not statistically significant. Antibody microarray showed ~ 1.3 fold reduction in N-terminal PARP fragment in response to Emodin in both lines (Fig. 2a). There was a significant (p < 0.01) decrease in PARP at both 36 and 48 h in DLD-1 cells. In contrast to DLD-1 there was an increase in PARP expression in COLO 201 at 36 h, and no change in PARP was observed at 48 h (Fig. 2c).

### Emodin disrupts mitochondrial outer membrane potential

Permeabilization of the mitochondrial outer membrane via a shift in membrane potential leading to release of intracellular proteins into the cytosol is a hallmark of intrinsic apoptosis. To investigate if Emodin-induced apoptosis affected mitochondria in human CoCa cells, mitochondrial outer membrane potential (MOMP) was assayed using JC-1 dye. The negative charge on mitochondria maintained by the membrane potential allows the JC-1 to oligomerize and accumulate in the mitochondrial matrix and fluorescently stain red. Monomeric JC-1 in the cytosol emits green fluorescence. So, JC-1 fluorescently stains both red (high delta/red elliptical gate) and green (low delta/green elliptical gate) in healthy cells. In apoptotic cells, JC-1 accumulation in the mitochondrial matrix is inhibited due to a collapse in membrane potential and they only fluorescently stain green (low delta/green elliptical gate) for the cytosolic JC-1. This allows apoptotic cells to be easily differentiated from healthy cells.

**Fig. 3:**
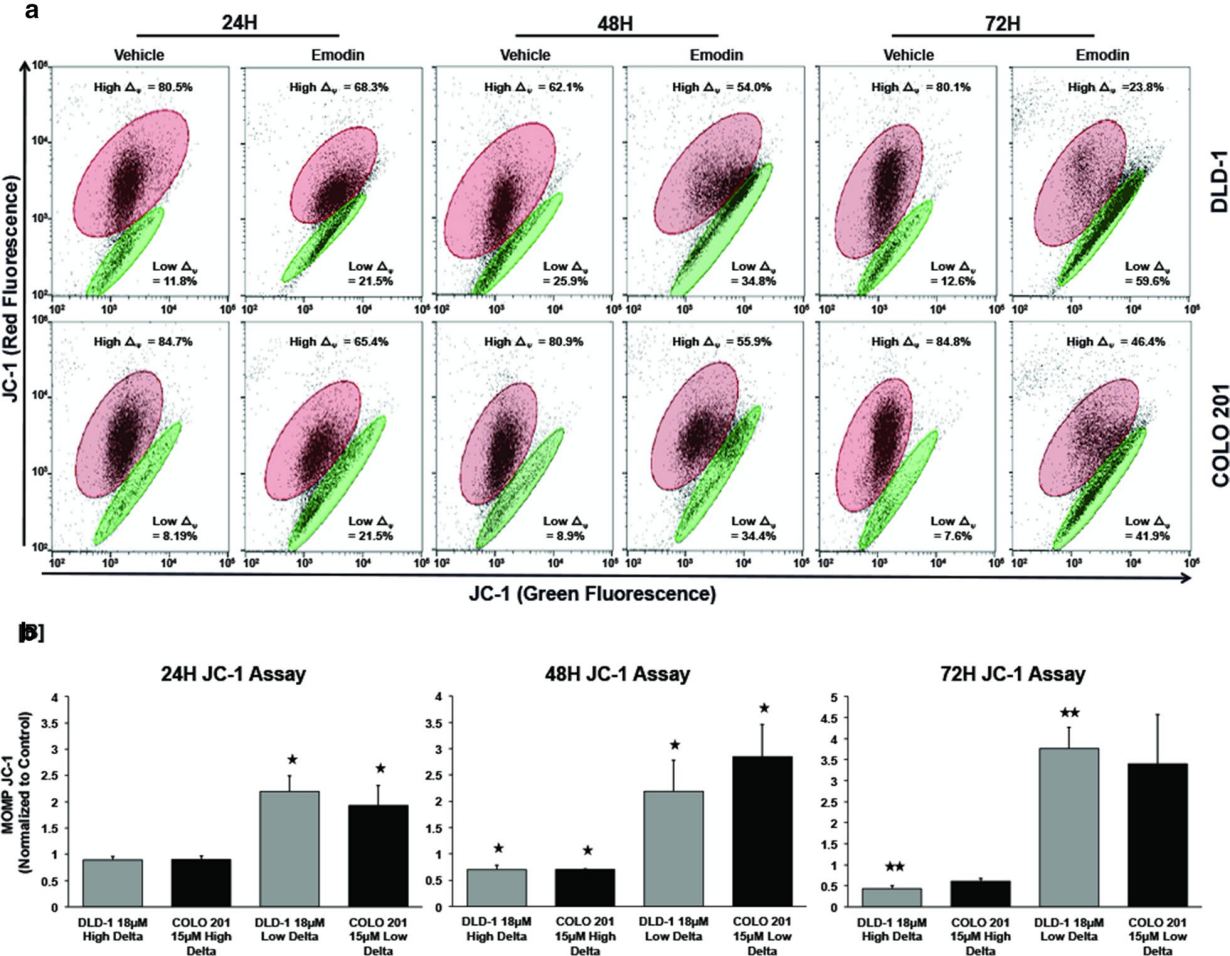
Effect of Emodin on the mitochondrial outer membrane potential. CoCa cells were treated with Emodin (DLD-1 ~ 18 μM; COLO 201 ~ 15 μM) for 24, 48, and 72 h. Vehicle treated cells were used as control. Mitochondrial outer membrane potential (ΔψM) was measured using JC-1 staining (**a**). Healthy cells with high Δψ_M_ form JC-1 aggregates showing red fluorescence (Red ellipse), while apoptotic cells with low Δψ_M_ exhibits green fluorescence (Green ellipse). Graphs represent the observed shift in the Δψ_M_ of DLD-1 and COLO-201 after Emodin treatment compared to vehicle control (**b**). Data represents mean ± SEM from three independent experiments. Asterisks (*) p < 0.05, (**) p < 0.01 show significant change after Emodin treatment compared with the control

Both CoCa cells showed a significant (p < 0.05) increase (DLD-1: 9.7%; COLO 201: 13.3%) in green fluorescent cells (JC-1 red-negative/JC-1 green-positive) after 24 h of Emodin treatment when compared to the control (Fig. 3a, b). At 48 h, healthy cell (JC-1 red-positive/JC-1 green-positive) populations significantly (p < 0.05) decreased (DLD-1: 8.1%; COLO 201: 25%) and apoptotic populations (JC-1 red-negative/JC-1 green-positive) significantly (p < 0.05) increased (DLD-1: 8.9%; COLO 201: 25.5%) compared to the control. At 72 h, healthy cell (JC-1 red-positive/JC-1 green-positive) populations significantly (p < 0.01) decreased (DLD-1: 56.3%; COLO 201: 40.4%) at after Emodin treatment and populations with mitochondrial damage (JC-1 red-negative/JC-1 green-positive) significantly (p < 0.01) increased (DLD-1: 47%; COLO 201: 34%) in both cell lines. The increase in JC-1 green-positive cells and decrease in mitochondrial JC-1 red-positive florescence implied loss in MOMP.

### Emodin regulates expression and localization of Bcl-2 family proteins

**Fig. 4:**
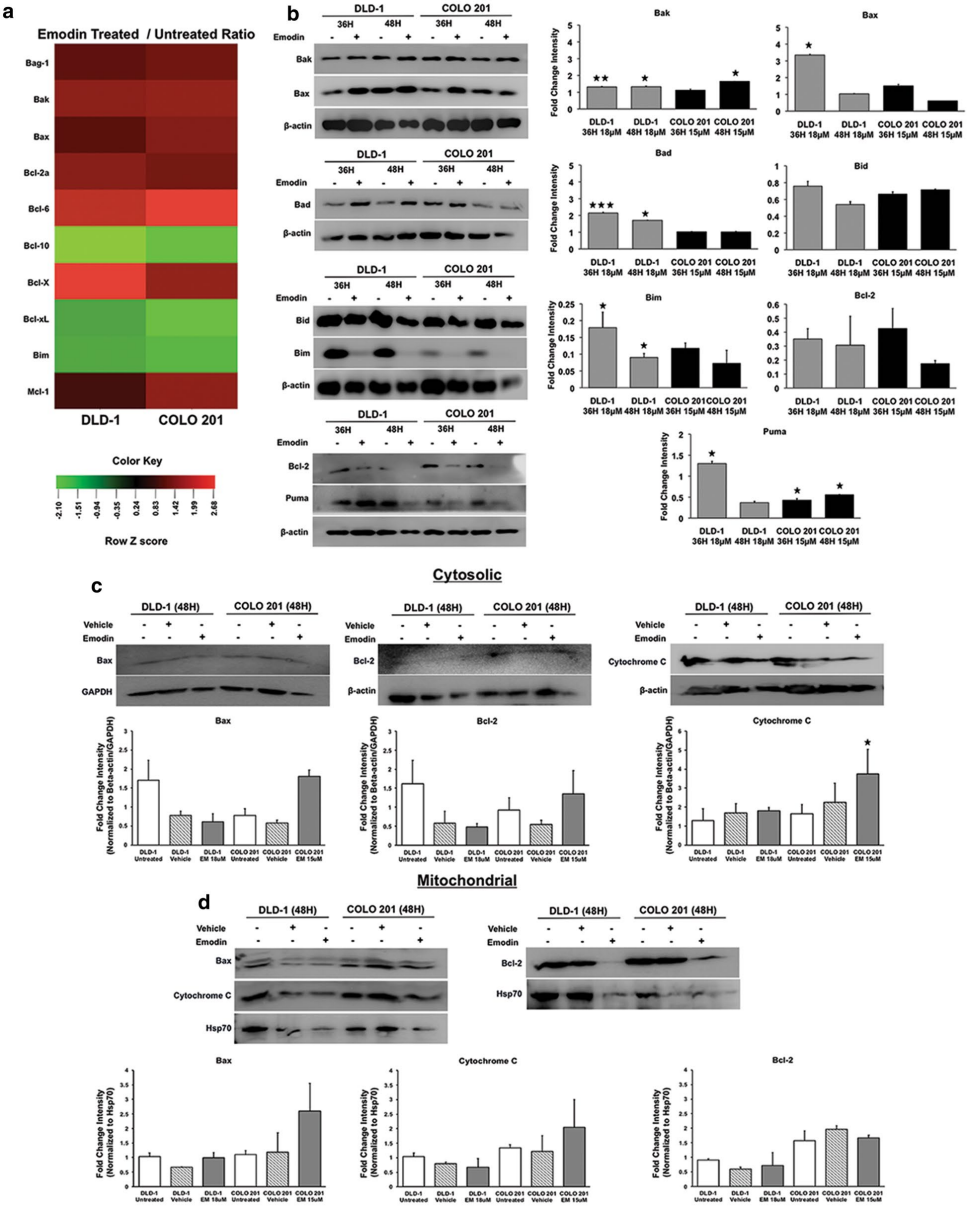
Effect of Emodin on expression and localization of BCL-2 family proteins. Heat map showing Emodin induced changes in Bcl-2 family apoptotic proteins was generated from the apoptosis antibody microarray data (**a**). Red represents increase while green represents decrease in protein expression, color intensity indicates on level of expression. Effect of Emodin treatment (DLD-1 ~ 18 μM; COLO 201 ~ 15 μM; 36 and 48h) on Bad, Bak, Bax, Bid, Bim, Bcl-2, and PUMA expression compared to vehicle control is shown by western blot (**b**). β-actin was used as a loading control. The quantitative bar graphs shown represent ratio of protein in Emodin treated versus vehicle-treated after normalization with β-actin band intensity. Effect of Emodin on cytosolic (**c**) and mitochondrial (**d**) distribution of  BAX, Bcl-2 and Cytochrome C is shown by western blot. Bar graphs show the quantitative distribution of the proteins in the two cellular fractions. β-actin or GAPDH was used as the loading control for cytosolic fractions and Hsp70 was used as a loading control for mitochondrial fractions. Densitometry analysis was done with respect to the intensities of loading control. All graphs represent the mean fold change intensity value ± SEM from three independent experiments; Asterisks (*) p < 0.05, (**) p < 0.01, (***) p < 0.001 show significant change after Emodin treatment compared with the control

Expression, localization, and binding of anti- and pro-apoptotic Bcl-2 family proteins are vital in regulating intrinsic apoptosis. An increase in Bax localization in ratio to Bcl-2 on the mitochondrial membrane can cause a leakage of mitochondrial  proteins into the cytosol leading to caspase activation and enhanced cell death. Bcl-2 family proteins expression was modulated by Emodin in CoCa cells (Fig. 4a). Pro-apoptotic Bcl-2 family proteins Bak (~ 1.4 fold) and Bax increased (~ 1 fold), while Bim levels decreased (~ 1.5-fold) in both CoCa cells after Emodin treatment. Anti-apoptotic Bcl-2 family members Bcl-2a, Bcl-X, Bcl-XL, and Mcl-1 were differentially modulated in both cell lines (Fig. 4a). Bag-1, Bcl-2a, Bcl-6, Bcl-X, and Mcl-1 protein expression increased after 48 h of Emodin treatment in both DLD-1 (Bag-1:1.0; Bcl-2: 1.4; Bcl-6: 1.8; Bcl-X: 2.0; Mcl-1: 0.8-fold) and COLO 201 (Bag-1: 1.0; Bcl-2: ~ 1.0; Bcl-6: 2.5; Bcl-X: ~ 1.4; Mcl-1: 1.4-fold) cells. Bcl-10 and Bcl-XL protein expression significantly decreased (Bcl-10 ~ 1.5; Bcl-XL ~ 1.2 fold) in both human CoCa cell lines. When these proteins were evaluated via western blot (Fig. 4b), pro-apoptotic Bak, and Bax were differentially modulated in both cell lines at 36 and 48 h after Emodin treatment. Bak levels significantly increased at 36 h (p < 0.01) and 48 h (p < 0.05) in DLD-1 cells. However, it significantly increased (p < 0.05) at 48 h in COLO-20. Bax increased at 36 h for both CoCa cells but only significantly for DLD-1 (p < 0.05). Pro-apoptotic proteins Bad and Puma were differentially modulated in both CoCa cells after Emodin treatment compared to control. Bad significantly increased at 36 h (p < 0.001) and 48 h (p < 0.05) in DLD-1 cells after treatment. PUMA levels significantly increased at 36 h (p < 0.05) for DLD-1 cells though it significantly decreased at both 36 h (p < 0.05) and 48 h (p < 0.05) in COLO 201 cells. Bim protein levels correlated with the array data as it decreased in both cell types however, the decrease was significant only in DLD-1 (p < 0.05) cells. Bid and Bcl-2 did not change significantly after Emodin treatment.

In cytosolic fraction, Bax protein levels decreased (~ 1.5-fold) in DLD-1 cells while increased (~ 1.5-fold) in COLO 201 cells after Emodin treatment compared to vehicle-treated cells. Cytosolic Bcl-2 expression correlated with Bax expression as it was decreased (0.1-fold) in DLD-1 cells and increased (~ 0.8-fold) in COLO 201 cells following Emodin treatment. Cytosolic cytochrome c levels increased in both cell lines after Emodin treatment. Increase was significant in COLO 201 cells (0.7-fold, p <0.05) (Fig. 4c). In mitochondrial fractions, Bax levels were elevated in both CoCa lines but COLO 201 cells had larger increase (1.0-fold). Mitochondrial Bcl-2 levels decreased (DLD-1: 0.6-fold; COLO 201: 0.4-fold) in both CoCa cell lines (Fig. 4c). Mitochondrial cytochrome c was differentially modulated in the two CoCa cell lines. DLD-1 cells showed a decrease while COLO 201 cells showed increase in mitochondrial localization of cytochrome c (Fig. 4d).

Total cytochrome c levels decreased (DLD-1: 0.98 fold; COLO 201: 1.35 fold) in CoCa cells after Emodin treatment when compared to the control (Fig. 2a). Western blot analysis (Fig. 2c) showed a decrease in total cytochrome c levels at both 36 h and 48 h in DLD-1 cells, but COLO 201 cells showed an increase in total cytochrome c at 48 h. These changes were  not significant (Fig. 2c).

### Emodin modulates signaling pathways involved in colon cancer growth and proliferation

**Fig. 5:**
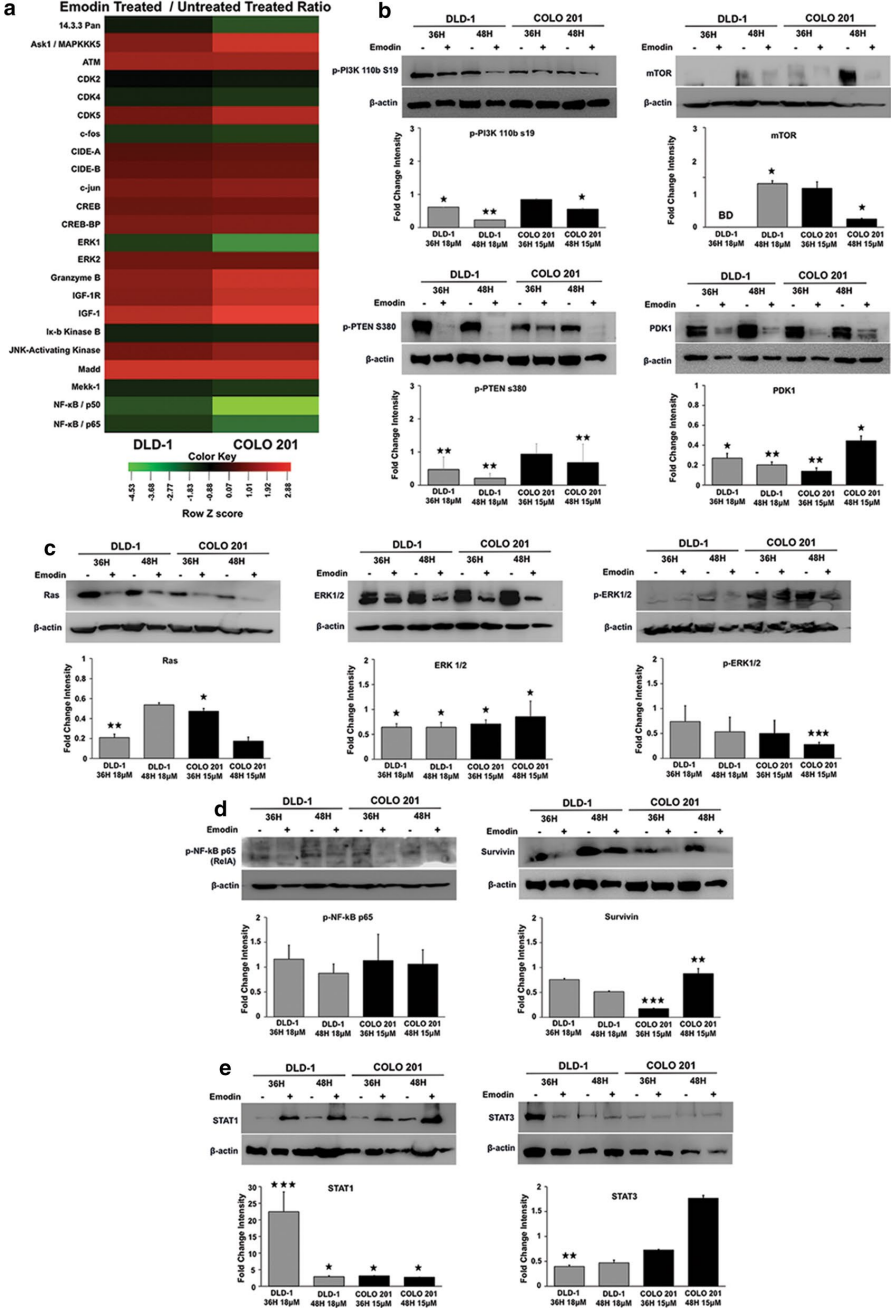
Emodin modulates growth and proliferation signaling pathways to promote apoptosis in colon cancer cells. Heatmap showing Emodin induced changes in cell survival related proteins was generated using apoptosis antibody microarray data (**a**). Each cell in the heatmap represents the fold change in protein expression in Emodin-treated CoCa cells compared to vehicle-treated control. Red represents increase while green represents decrease in protein expression, and intensity of color depends on level of expression. **e** Effect of Emodin treatment (DLD-1 ~ 18 μM; COLO 201 ~ 15 μM; 36 and 48h) on expression of key signaling molecules of PI3K/AKT, MAPK/ERK, NF-κβ and Jak/STAT pathways compared to vehicle control is shown by western blot (**b**–**e**). β-actin was used as the loading control. The quantitative bar graphs shown represent ratio of protein in Emodin treated versus vehicle-treated after normalization with β-actin band intensity. ‘BD’ represents below detection. All graphs represent the mean fold change intensity value ± SEM from three independent experiments; Asterisks (*) p < 0.05, (**) p < 0.01, (***) p < 0.001 show significant change after Emodin treatment compared with the control

Antibody microarray analysis showed a modulation of MAPK/ERK and NF-κβ pathways involved in CoCa cell growth and proliferation in response to Emodin (Fig. 5a). Expression of MAPK/ERK signaling components ASK1, ERK1, ERK2, and MEKK-1 were modulated by Emodin treatment. ASK1 (MAP3K5) expression increased (DLD-1: 1.2-fold; COLO 201: 1.8-fold) in both cell lines in response to Emodin treatment. ERK1 expression decreased in both CoCa cells with a higher decrease (1.5-fold) in COLO 201 than DLD-1. Emodin treatment decreased ERK2 and MEKK-1 expression (~ 0.4-fold) in both cell lines compared to control. Downstream molecules activated by MAPK/ERK signaling, such as JNK activating kinase and MADD were also assessed after Emodin treatment. JNK activating kinase had relatively no change in expression (0.14-fold) in DLD-1 cells and moderately increased (1.2-fold) in COLO 201 cells while MADD protein expression was upregulated (~ 2.3-fold) after Emodin treatment in both cell lines (Fig. 5a). Expression of MAPK/JNK pathway molecules such as Ras, MAPK/ERK½, and pMAPK/ERK½ were confirmed via western blot (Fig. 5c). Ras expression significantly decreased in both cell lines after Emodin treatment at 36 h (DLD-1: p < 0.01; COLO 201: p < 0.05). MAPK/ERK1/2 expression significantly decreased at both 36 (p < 0.05) and 48 h (p < 0.05) in both cell lines following treatment. Levels of pMAPK/ERK½ (Thr202/Tyr204) also decreased at 36 and 48 h in both cell lines but this decrease was only significant in COLO 201 (p < 0.001) at 48 h.

Expression of NF-κβ signaling components NF-κβ p50, NF-κβ p65 (Rel A), and IKΚβ were modulated by Emodin treatment (Fig. 5a, d). Emodin treatment decreased NF-κβ p50 expression in both cell types. The decrease was more in the COLO 201 ﻿(2.1-fold) cells at 48 h (Fig. 5a). NF-κβ p65 (RelA) expression decreased 0.8-fold following Emodin treatment in both cell types as observed by antibody array. However, NF-κβ p65 (RelA) Ser536 phosphorylation did not change significantly after Emodin treatment (Fig. 5d). IΚΚβ expression decreased marginally (0.4-fold) in the array when compared to control (Fig. 5a). Emodin also decreased survivin expression in CoCa cells. The decrease was significant at both 36 (p < 0.001) at 48 h (p < 0.01) in COLO 201 cells (Fig. 5d).

Phosphorylation of PI3K 110b at S19, a site necessary for the activation for PI3K, was significantly modulated by Emodin (Fig. 5b). In DLD-1 cells, PI3K expression in response to Emodin significantly decreased at 36 h (p < 0.05) and 48 h (p < 0.01), while it was significantly decreased only at 48 h (p < 0.05) in COLO 201 cells. PDK1 expression significantly decreased at 36 h (DLD-1: p < 0.05; COLO 201: p < 0.01) and 48 h (DLD-1: p < 0.01; COLO 201: p < 0.05) in CoCa cells. Phosphorylation of PTEN at S380, a negative regulator of PI3K/AKT signaling, significantly decreased at both 36 h (DLD-1: p < 0.01) and 48 h (p < 0.01) in CoCa cell lines after Emodin treatment. mTOR expression also significantly (p < 0.05) decreased 48 h after Emodin treatment. This information shows that Emodin regulates PI3K/AKT signaling in CoCa cells. Emodin also regulates STAT signaling pathways in CoCa cells (Fig. 5e). STAT1 significantly increased at 36 h (DLD-1: p < 0.001; COLO 201: p < 0.05) and 48 h (p < 0.05) in both cell lines. STAT3 expression decreased significantly at 36 h (p < 0.01) in DLD-1 cells whereas in COLO 201 cells showed an increase at 48 h when compared to control.

## Discussion

Colon cancer (CoCa) is a leading cause of cancer-related deaths worldwide [29, 30]. Though ~ 95% of CoCa cases are considered to be sporadic, early detection via routine health screenings has been effective in reducing CoCa risk by up to 90% in patients 50 years or older [1–3]. Unfortunately, a great number of patients succumb to advanced stage disease before adequate treatment can be received [21, 22]. Treatment for advanced stage CoCa primarily consists of corrective surgery in combination with chemotherapy. Adverse side effects such as chemo-resistance and micro-metastasis have been associated with these treatment options that show only a 14% 5-year survival rate [4–7]. Due to these complications, there continues to be a need to explore new avenues to treat CoCa more effectively with minimal toxicity to patients.

Emodin, a naturally occurring anthraquinone, has a wide range of pharmacological properties that make it an attractive candidate for cancer therapeutics such as its ability to induce apoptosis, cell cycle arrest, and inhibit DNA synthesis [20–28]. In cancers affecting the digestive tract, Emodin decreased metastasis, reduced survival signaling, and enhanced antitumor effects of other chemotherapeutic agents [5, 31–36]. Our data shows Emodin reduced the proliferation of CoCa cells in a concentration and time-dependent manner. This inhibition was achieved at relatively low concentrations (~ 20 μM) while toxicity to normal colon epithelium was relatively low. Reduced viability was mainly due to Emodin-induced apoptosis of CoCa cells. Though inhibition of proliferation has been documented, there is a lack of information regarding the molecular changes associated with Emodin induced apoptosis in CoCa. We provide a deeper molecular perspective on how Emodin induces apoptotic cell death in CoCa. Our data shows Emodin treatment induces apoptosis in CoCa by differentially modulating the expression of extrinsic and intrinsic apoptotic molecules, cell survival signaling, and the localization or activity of Bcl-2 family of proteins.

Bcl-2 family proteins are key regulators of intrinsic apoptosis as their expression, localization, and binding has been associated with mitochondrial dysfunction, leakage of cytochrome c, and downstream apoptotic activation [22, 32, 37–39]. Emodin’s ability to modulate both anti- and pro-apoptotic Bcl-2 family proteins during apoptosis has been observed in several cancer models. However, information on regulation of the expression, localization, and function of these proteins in CoCa apoptosis induced by Emodin is limited. We observed a direct modulation of Bcl-2 family proteins level and localization during Emodin-induced apoptosis. Total cell expression of pro-apoptotic Bcl-2 family proteins Bak and Bax were differentially increased in CoCa models compared to Bcl-2 levels. Analysis of cytosolic and mitochondrial fractions of CoCa cells revealed an increase in the levels of Bax in COLO 201 cells in contrast to anti-apoptotic Bcl-2 protein levels. Previous studies have indicated that increase in pro-apoptotic proteins Bax and Bak with decreased levels of anti-apoptotic proteins like Bcl-2 in both total and mitochondrial fractions are a major driving force in mitochondrial dysfunction and subsequent apoptosis in some anti-cancer treatments [16, 25, 29, 31–33]. Our data further supports these observations as Emodin regulated Bcl-2 family protein levels and localization that could lead to the observed loss of MOMP, increased cytosolic cytochrome c levels, and apoptosis.

Small subsets of pro-apoptotic Bcl-2 family proteins, known as BH3-only, were also differently modulated during Emodin-induced apoptosis. BH3-only proteins enhance apoptosis by targeting and inhibiting anti-apoptotic Bcl-2 proteins while enhancing pro-apoptotic Bcl-2 protein localization and potency [31]. Emodin has been shown to increase the expression and activity of this protein subset in other cancer models, but such effect of Emodin on CoCa cells has not been fully elucidated. We found that Emodin differentially modulated the expression levels of BH3-only proteins Bad, Bid, Bim, and Puma in these CoCa cells during apoptosis. The expression levels and function of these proteins are known to be stimulated by tumor suppressor p53, which is mutated in both cell types we used. Our data showed that Emodin treatment increases p53 expression. Increased Bad protein expression seemed to facilitate pro-apoptotic BH3-only activity in DLD-1 cells while Bid, Bim, and Puma levels were reduced. It is known that decreased caspase-8 activity and low p53 expression reduce Bid and Puma activation in CoCa cells. Bim was reduced during Emodin-induced apoptosis in CoCa cells. Reduced Bim expression is a feature of some cancers and contributes to an apoptosis-resistant phenotype in advanced stage disease [31, 40]. Though no studies have reported this phenomenon, we postulate that although Emodin treatment reduces Bim, it may be compensated by up-regulation of other pro-apoptotic Bcl-2 proteins in CoCa. Our data indicates that Emodin’s ability to differentially regulate BH3-only protein expression maybe a key feature in its ability to induce CoCa apoptosis.

**Fig. 6:**
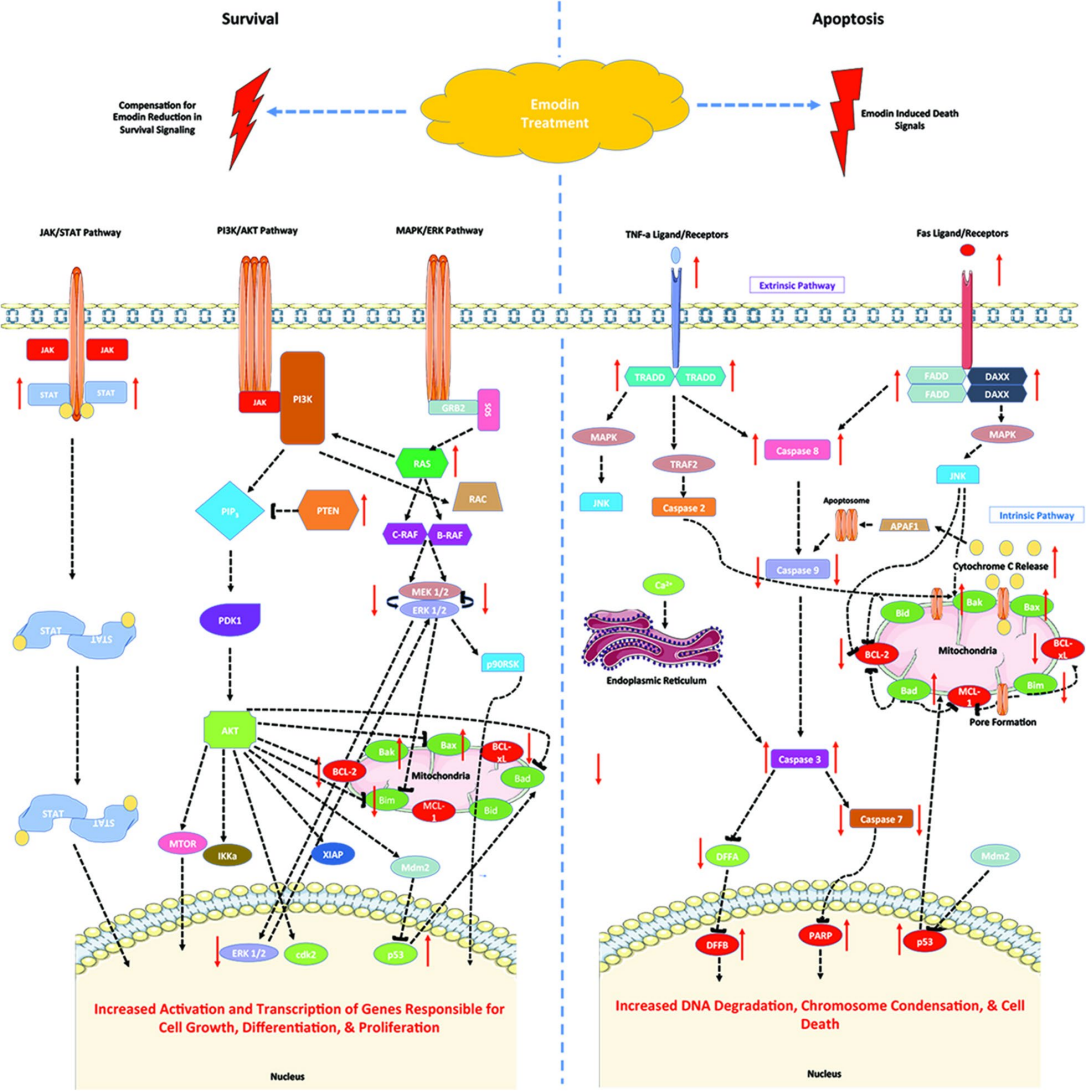
Schematic diagram showing effect of Emodin on colon cancer apoptosis and survival. Emodin modulates JAK/STAT, PI3K/AKT and MAPK/ERK pathways regulating the genes responsible for cell growth, differentiation and proliferation. Emodin regulates intrinsic and extrinsic pathways of apoptosis in CoCa cells by modulating caspase activation and localization and expression of Bcl-2 family proteins

Cancer cells steer cell machinery to gain self-sufficiency in proliferation while evading death signaling. Dysregulated growth and survival signaling can modify the expression and potency of apoptotic molecules during apoptosis. Previous studies have shown Emodin that modulated growth and survival signaling pathways associated with cancer proliferation and evasion of apoptosis [26, 28, 38, 41–44]. Our data shows Emodin reduces several regulatory components involved in MAPK/JNK, PI3K/AKT, NF-κβ, and STAT pathways associated with apoptotic functions of Bcl-2 family proteins. MAPK/JNK pathway has been shown to regulate the activity and levels of Bcl-2 family proteins post-translationally by phosphorylating serine residues on apoptotic proteins like Bim. Phosphorylation by MAPK (ERK1/2) can induce Bim ubiquitination and reduced binding to anti-apoptotic Bcl-2 or enhance its stability [16, 39]. MAPK (ERK1/2) and phosphorylated MAPK protein were reduced after Emodin treatment of CoCa cells, which correlated to reduced Bim expression. C-Jun (JNK) also phosphorylates Bim inducing its cleavage from dynein complexes and binding to other Bcl-2 family proteins. Reduced expression of these MAPK/JNK signaling components may reduce Bcl-2 family pro-apoptotic functions. It could lead to the reduction in expression of these proteins as observed after Emodin treatment as summarized in Fig. 6. Similar correlations to Bcl-2 family proteins level were observed in the PI3K/AKT pathway during Emodin-induced apoptosis. It reduced phosphorylation of catalytic complex 110b of PI3K and pPTEN S380. Reduced 110b expression can limit PI3K activation and downstream activation of mTOR and PDK1. Aberrant mTOR signaling promotes AKT activation leading to increased proliferation and inhibition of cell death in many cancers. Studies indicate Emodin decreases the expression and function of mTOR [33, 45–49], and we observed similar results as mTOR expression was significantly decreased in CoCa cells. PDK1 inhibition impairs cell growth and increases cell death under hypoxia in cancer. Its expression decreased in CoCa following Emodin treatment, correlating with reduced PI3K/AKT signaling during CoCa apoptosis. PI3K/AKT signaling also inhibits the activity and translocation of pro-apoptotic Bax to the mitochondrial membrane and represses downstream transcription of Bim via FOXO3a [26, 39–43]. Our data suggests, as shown in Fig. 6, Emodin induces cell death in CoCa cells by reducing PI3K/AKT signaling that otherwise protects cancer from death.

Effect of Emodin on NF-κβ signaling in cancer, including CoCa, is well documented. NF-κβ signaling regulates inflammation, stress, and B cell development, but also has been associated with function of Bcl-2 family proteins. NF-κβ signaling is regulated by the binding and inhibition of Ikβ proteins such as IKΚβ that forms a complex sequestering NF-κβ. In our study, Emodin decreased the expression of NF-κβ proteins-NF-κβ p50, NF-κβ p 65 (Rel A), and IKΚβ. Studies have shown that NF-κβ signaling enhances transcription and anti-apoptotic potency of Bcl-2 family proteins by increasing transcription of gene promoter site Bcl-2 p2 [41, 50]. Thus decreased NF-κβ expression correlates to reduced Bcl-2 expression in Emodin induced apoptosis in CoCa.

Signal transducers and activators of transcription (STATs) regulate growth, survival, and differentiation. Correlations have been made with STAT signaling and anti-apoptotic Bcl-2 expression, however, such correlations have not been investigated for Emodin induced apoptosis [38, 42]. Increased STAT1 expression after Emodin treatment correlated with increased pro-apoptotic Bcl-2 family expression except for Bim. This data was interesting as increased expression of pro-apoptotic proteins such as Bax have been shown to increase STAT activation as a result of ROS generation and increased TNF-α mediated cell death [42]. TNF-α and Bax expression did increase during Emodin-induced apoptosis, which could correlate to increased STAT1 expression. Overall, our data shows that Emodin modulates survival signaling by regulating the expression and function of Bcl-2 family proteins during CoCa apoptosis (Fig. 6).

## Conclusion

Emodin affects anti- and pro-survival signaling cascades essential in cell proliferation, differentiation, survival and apoptosis in CoCa. Emodin also regulates expression and localization of Bcl-2 family proteins. It could do so by regulating MAPK/JNK, PI3K/AKT, NF-κβ and STAT molecular signaling pathways that dictate Bcl-2 family protein functions. In all, Emodin could be a potential treatment option for CoCa as it differentially regulates many of the key apoptotic and survival signaling mechanisms.

## Abbreviations

CoCa: colon cancer
MTT: 3-(4,5-dimethylthiazol-2-Yl)-2,5-diphenyltetrazolium bromide
MOMP: mitochondrial outer membrane potential
